# Effect of Thyroxine on the Structural and Dynamic Features of Cardiac Mitochondria and Mitophagy in Rats

**DOI:** 10.3390/cells12030396

**Published:** 2023-01-21

**Authors:** Natalya Venediktova, Ilya Solomadin, Vlada Starinets

**Affiliations:** Laboratory of Mitochondrial Transport, Institute of Theoretical and Experimental Biophysics, Russian Academy of Sciences, 142290 Pushchino, Russia

**Keywords:** mitochondrial dysfunction, energy metabolism, thyroid hormones, quality control system, mitophagy

## Abstract

This work investigated the effect of thyroxine on the biogenesis and quality control system in rat heart mitochondria. In hyperthyroid rats, the concentrations of free triiodothyronine and thyroxine increased severalfold, indicating the development of hyperthyroidism in these animals. The electron microscopy showed 58% of cardiac mitochondria to be in a swollen state. Some organelles were damaged and had a reduced number of cristae. Multilamellar bodies formed from cristae/membranes were found in the vacuolated part of the mitochondria. The hyperthyroidism caused no changes to mitochondrial biogenesis in the investigated animals. At the same time, the levels of mitochondrial dynamics proteins OPA1 and Drp1 increased in the hyperthyroid rats. The administration of thyroxine to the animals led to a decrease in the amount of PINK1 and Parkin in heart tissue. The data suggest that excess thyroid hormones lead to changes in mitochondrial dynamics and impair Parkin-dependent mitophagy in hyperthyroid rat heart.

## 1. Introduction

The thyroid gland produces two types of iodothyronines—tetraiodothyronine (thyroxine) and triiodothyronine—which play a central role in the regulation of development, differentiation, growth, and metabolism in most higher organisms. Although thyroxine (T_4_) is the main product of the thyroid gland, a biologically active hormone is triiodothyronine (T_3_). The biological activity of triiodothyronine is largely determined by its intracellular concentration [1]. Depending on the content of thyroid hormones (THs), the organism’s metabolism can be accelerated or slowed down. As TH can enhance calorigenesis by stimulating cellular respiration, mitochondria are a key object in studying the mechanisms of hyperthyroidism (HT) development.

Mitochondrial dynamics and mitophagy are the basic processes underlying mitochondrial homeostasis [2]. Fusion and fission determine the architecture of a cell’s mitochondrial population and affect almost all aspects of mitochondrial functions, including respiration, calcium capacity, and apoptosis [3,4,5]. Mitochondrial fusion enables the distribution of metabolites, enzymes, and products of mitochondrial genes throughout the mitochondrial compartment. This optimizes the functionality of mitochondria and counteracts the accumulation of mitochondrial mutations in aging. Mitochondrial fission plays an important role in the removal of damaged organelles by autophagy [6]. It is mainly mediated by dynamin-related protein 1 (Drp1), an evolutionarily conserved GTPase in eukaryotic cells that is normally distributed in the cytosol. Mitochondrial fusion is a two-step process mediated by outer mitochondrial membrane proteins mitofusins (Mfn1 and Mfn2) and inner mitochondrial membrane protein optic atrophy 1 (OPA1) [7]. To date, only a few articles on the effects of TH on fusion and fission processes have been reported in the literature [8].

Mitophagy is a highly conserved process in which damaged mitochondria are engulfed by two-membrane autophagosomes and transferred to lysosomes to be degraded and recycled. There are several different mechanisms of mitophagy, of which the PINK1–Parkin pathway is the most investigated. When mitochondrial depolarization is irreversible, PTEN-induced kinase 1 (PINK1) is recruited to the outer mitochondrial membrane and stabilizes on it. Parkin then ubiquitinates SQSTM1/p62 receptor protein (sequestosome 1), which attracts mitochondria to the isolating membranes of the autophagosome using LC3 protein [9]. The deletion of PINK1 or Parkin can interfere with mitophagy and simultaneously lead to mitochondrial elongation [10]. PINK1–Parkin independent mitophagy employs several receptors: BNIP3 (Bcl-2/adenovirus E1B protein interacting with protein 3), Nix/BNIP3L (Nip3-like protein X), FUNDC1 (FUN14 domain-containing protein 1), and cardiolipin. They are localized in mitochondria and interact with LC3, attracting autophagosomes to damaged mitochondria. In addition, damaged mitochondria can also undergo direct remodeling to form mitochondrial vesicles (MVs) or mitochondrial spheroids [11].

Mitochondrial dynamics, biogenesis, and mitophagy are among the processes that can be strongly regulated by TH. There is no universal molecular model to explain the various actions of TH. Even the administration of TH to one species of an animal under study can cause different responses in different organs. For this reason, it is difficult to obtain an unambiguous answer as to the effect of TH on the metabolism as a whole. We investigated the effect of TH on the regulation and quality control of mitochondria and mitophagy in animal heart on a hyperthyroidism model. The results of this work can provide not only new basic knowledge regarding the functioning of mitochondria in conditions of increased TH concentration but also new prospects for the development of therapeutic strategies to correct this pathology and some other diseases associated with altered mitochondrial bioenergetics.

## 2. Materials and Methods

### 2.1. Animals

The research was conducted on 3–3.5 month old Wistar male rats (weight: 210–230 g). The animals were randomly divided into control and experimental groups (*n* ≥ 5 for each group). Hyperthyroidism was induced by intraperitoneal injection of L-thyroxine at a dose of 200 μg per 100 g of animal weight for 7 days. The control rats were injected with an equal volume of saline. The rats were sacrificed by cervical dislocation at the same time. The plasma levels of free T_3_ and T_4_ were determined by an enzyme-linked assay (X-3962 T_4_ free; X-3970 T_3_ free, Vector-BEST, Novosibirsk, Russia).

### 2.2. Electron Microscopy

Samples of the heart tissue for the transmission electron microscopy (TEM) imaging were taken from decapitated animals and fixed in a 2.5% glutaraldehyde solution in 0.1 M phosphate-buffered saline (PBS, pH 7.4) for 2–3 h [12]. The preparations were examined and photographed using a JEM-2000 FXII (JEOL Ltd., Tokyo, Japan). The morphometric analysis of the images was carried out using the Image J 1.52n software. Approximately 35–50 electron micrographs for each individual animal were studied (n = 4). 

### 2.3. Quantification of the mRNA Expression of Mitochondrial Dynamics, Mitochondrial Biogenesis, and Mitophagy Genes Using Quantitative Real-Time PCR

The total RNA from control rat (CR) and hyperthyroid rat (HR) heart samples (approximately 100 mg) was obtained using ExtractRNA reagent (Evrogen, Moscow, Russia) in accordance with the manufacturer’s protocol. The RNA preparation obtained was treated with RNase-free DNase I (Thermo Fisher Scientific, Waltham, MA, USA). Real-time PCR was performed by the QuantStudio 1 amplifier (Thermo Fisher Scientific, Waltham, MA, USA) using the qPCRmix-HS SYBR kit (Evrogen, Moscow, Russia) with SYBR Green II as a fluorescent intercalating dye. The gene-specific primers were chosen and analyzed using Primer-BLAST [13] (the oligonucleotide sequences are given in Table 1). The relative level of the expression of each gene was normalized by the level of Actb mRNA; the results obtained were quantified by the comparative CT method [14]. The ∆∆Ct was calculated by the formula ∆∆Ct = ∆Ct (experiment) − ∆Ct (control); each value of ∆Ct was calculated as ∆Ct = Ct (investigated gene) − Ct (housekeeping gene) [14].

### 2.4. Immunoblotting Analysis

Th total protein extracts were prepared from 10 to 15 mg of the deep-frozen heart tissue. To maintain the extract integrity and function, we used Complete Protease Inhibitor Cocktail (P8340, Sigma-Aldrich, St. Louis, MO, USA), Phosphatase Inhibitor Cocktail II (ab201113, Abcam, Waltham, MA, USA), PMSF (1 mM), EGTA (1 mM), and EDTA (1 mM). The proteins were isolated using a 1xRIPA buffer (ab156034, Abcam, Waltham, MA, USA). The concentration of the protein was determined by the Lowry method [15]. The samples (30–50 µg) were diluted in Laemmli buffer, separated by 12.5% SDS-PAGE, and transferred to a nitrocellulose membrane (Thermo Fisher Scientific, Wilmington, USA) [12]. The relative levels of the detected proteins were visualized using a C-DiGit Blot Scanner (LI-COR Biotechnology, Lincoln, Nebraska, USA) and normalized to the appropriate loading control. The antibodies used: (ab56788) anti-DRP1 (1:1000), (ab119685) anti-OPA (1:1000), (ab124773) anti-Mitofusin2 (1:1000), (ab54481) anti-PGC1a (1:1000), (cs2132) anti-Parkin (1:1000), (Affinity Biosciences, DF7742) anti-PINK1 (1:250), (Affinity Biosciences, AF5384) anti-SQSTM1/p62 (1:250), (Affinity Biosciences, DF8163) anti-BNIP3L (1:250), and (cs12741) anti-LC3A/B (1:1500). The anti-GAPDH (ab181602) and anti-tubulin (1:2000) (ab4074) antibodies were used as a loading control.

### 2.5. Statistical Analysis

The data were analyzed using the GraphPad Prism 6 and Excel software and presented as the means ± standard errors of the mean (SEM) of 5-15 independent experiments in each group. We used the Shapiro–Wilk normality test of GraphPad Prism to determine the Gaussian distribution in our data sets. If the data fit the normal distribution, the statistical significance of the differences between the experimental groups was analyzed with two-tailed *t*-test. The data for the Western blot analysis were estimated using the Mann–Whitney test. 

## 3. Results

### 3.1. Characterization of the Animals with Experimentally Induced Hyperthyroidism

The standard test to determine thyroid pathologies is to measure the concentration of T_3_ and T_4_ in blood serum. The concentration of free T_3_ and T_4_ in HR increased by approximately two and three times, respectively (Table 2). The administration of thyroxine also caused a decrease in HR body weight but increased heart weight (Table 2). The body weight gain in the HR animals decreased by more than two times. An increase in the heart/body weight ratio in HR was indicative of a cardiac hypertrophy induced by T_4_.

### 3.2. Ultrastructural Features of Heart Tissue in the Control and Hyperthyroid Rats

**Figure 1:**
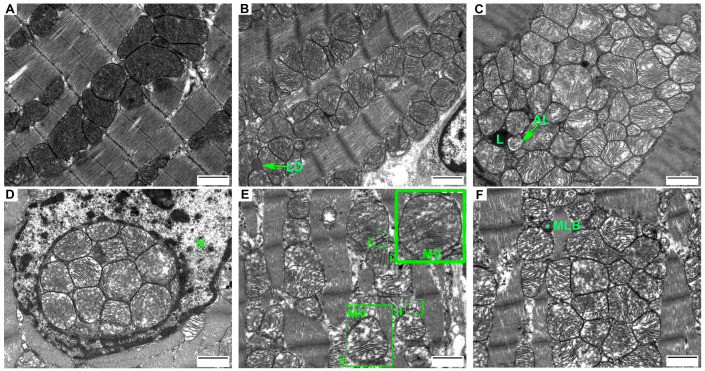

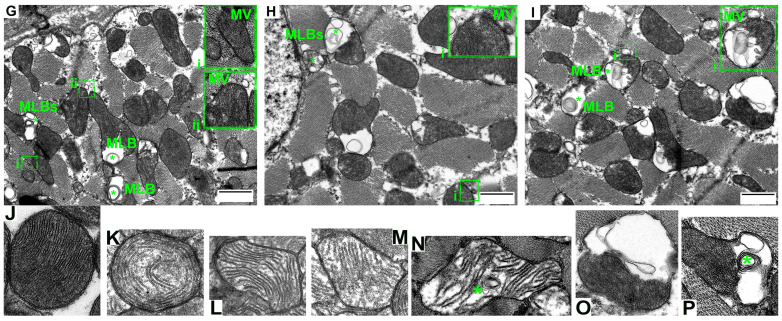
Representative micrographs of heart tissue in the control (**A**–**C**) and in experimental hyperthyroidism (**D**–**I**). Asterisk, multilamellar bodies (MLBs); MVs, mitochondrial vesicles; N, nucleus; L, lysosome; AL, autolysosome; LD, lipid droplet. Scale bar, 1 μm. (**J**–**P**) Electron micrographs of the morphological profile of encountered mitochondria: (**J**) well-packed, intact, and organized membranes and cristae; (**K**) mitochondria with concentric “onion-shaped” cristae; (**L**,**M**) swollen mitochondria with a slight decrease in the electron density of the matrix and irregular cristae; (**N**) swollen mitochondria with MLBs; (**O**) damaged mitochondria, matrix vacuolization, and cristae are almost not defined; (**P**) damaged mitochondria with MLBs. The number of mitochondria analyzed in each group varied from 600 to 700 (n = 4 in each group).

Earlier, we showed a change of oxygen uptake in HR heart mitochondria with a decrease in the respiratory control ratio when using different substrates. The development of oxidative stress has also been observed [16]. These changes can occur in experimental animals due to the accumulation of dysfunctional mitochondria in a state of hyperthyroidism. This was the reason for us to study the ultrastructure of heart tissue in control and hyperthyroid animals. Figure 1A–C,J clearly show parallel myofibrils having a structure typical of animal heart tissue. The sarcoplasmic reticulum consisted of short cisternae, vesicles, and tubules located near the nucleus, between mitochondria or bundles of myofibrils. The nuclei were of rounded shape and were oriented along the direction of muscle fibers. The moderately wavy nuclear membrane formed single invaginations. Lipid droplets, lysosomes, and autolysosomes occurred in the cytoplasm of myocytes (Figure 1B,C). The CR heart mitochondria were oval or elongated, with well-packed numerous cristae and an electron-dense matrix. As a rule, the cristae were parallel to one another but were sometimes concentric onion-like (Figure 1C,K). The mitochondria were visualized near the nuclei, between the myofibrils and under the sarcolemma. The nuclei of the CR cardiomyocytes were of rounded or oval shape, with smooth nuclear envelopes or sometimes with single invaginations in them.

HR also had myofibrillar, sarcolemmal, and nuclear mitochondria. In Figure 1E–I, the cytoplasm of cardiomyocytes is seen to be edematous. The ordered arrangement of sarcoplasmic-network tubules is disturbed by their fragmentation and swelling. HR had cardiomyocytes with swollen and hypertrophied mitochondria (58%), which were closely adjacent to one another and featured a partial disorientation of cristae (Figure 1E,F,M and Table 3). These organelles were noted to have a decrease in the electron density of the matrix, but the number of cristae was commensurate with that in CR mitochondria. Some organelles (13%) were damaged and had vacuoles that divided the mitochondrion into two parts (Figure 1H,I and Table 3). Multilamellar bodies formed from the cristae/membranes of these organelles often occurred in the vacuolated part (Figure 1F–I and Table 3) [17]. Yet another feature of HR was the formation of vesicles (MVs) on the outer mitochondrial membrane of 84.4 ± 16 nm in size (Figure 1E,G–I). Figure 1D shows a mitochondrial cluster surrounded by chromatin inside the HR nucleus. The HR cardiomyocyte nuclei were of different shapes (from rounded to cylindrical) and had a membrane that formed three or more invaginations. In some cases, chromatin condensation in the nuclei of HR cells was observed. 

Morphometric analysis of the cardiac electron micrographs showed a tendency to decrease in the number of HR heart mitochondria (Table 3). At the same time, the number of damaged mitochondria (vacuolization of the matrix with MLBs) in the HR was five times greater than in the CR (Table 3).

**Figure 2:**
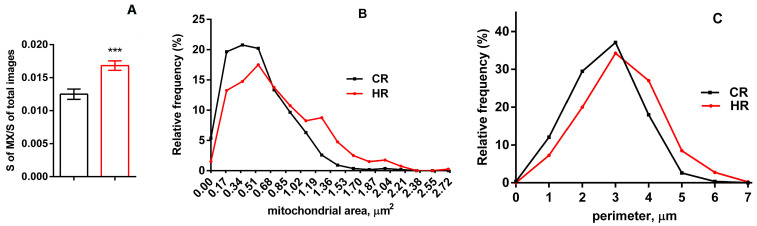
Morphometric parameters of heart mitochondria from the experimental rats. (**A**) The ratio of the mitochondrial area to that of the total image area (48 µm^2^). The number of examined images in each group was approximately 50. (**B**) A histogram of the distribution of the mitochondrial area in the groups. (**C**) A histogram of the distribution of the mitochondrial perimeter in the groups. The number of mitochondria analyzed in each group varied from 600 to 700. CR, control; HR, hyperthyroidism. *** *p* < 0.01, as compared with the control data (n = 4).

The analysis of the cardiac preparations revealed an increase in the mitochondrial area in the HR heart (Figure 2A). It is seen in Figure 2B that the number of organelles of a 0.1 µm^2^ area decreased by five times; of a 0.55 µm^2^ area, two times. The same pattern was observed with respect to the perimeter; in HP, the number of mitochondria of a perimeter of up to 1 µm decreased by six times; of a perimeter of 1–3 µm, by two times (Figure 2C). At the same time, the number of mitochondria of an area exceeding 1 µm^2^ and a perimeter of 5 µm increased significantly (by two and three times, respectively) (Figure 2B,C).

### 3.3. Gene Expression Differences in Heart Tissue of the Control and Hyperthyroid Rats

**Figure 3:**
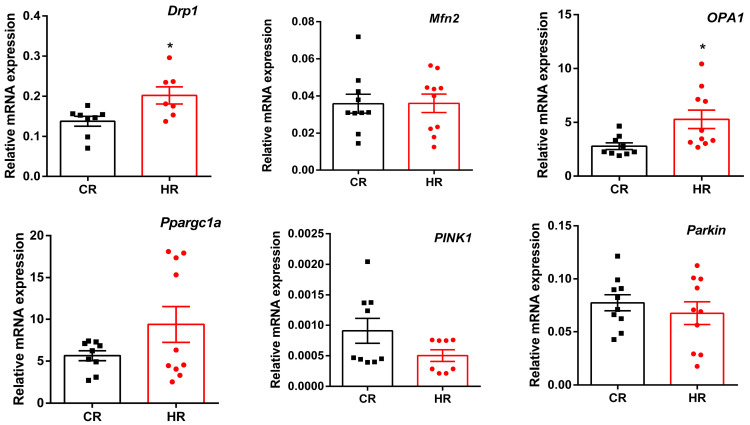
Quantitative real-time PCR analysis of the mRNA expression of mitochondrial dynamics, biogenesis and mitophagy genes in heart of experimental animals. CR, control; HR, hyperthyroidism. * *p* < 0.05, as compared with the control data (n = 8).

Previously, we showed a 15–29% decrease in the respiratory control ratio, as well as the development of oxidative stress, in HR mitochondria [16]. These events could be associated with changes in mitochondrial biogenesis and dynamics occurring in the animals after the development of hyperthyroidism. To assess the effect of TH on these processes, we measured the expression of genes encoding proteins responsible for mitochondrial fusion (*Mfn2* and *OPA1*) and fission (*Drp1*), mitochondrial biogenesis (*Ppargc1a*) and mitophagy (*PINK1* and *Parkin*). It turned out that in HR the expression level of *Ppargc1a* tended to increase (Figure 3). The level of the mRNA expression of the *Mfn2* gene did not change; at the same time, the expression of two other genes (*OPA1* and *Drp1*) involved in mitochondrial dynamics increased in HR heart by 1.9 and 1.5 times, respectively. We also noted a tendency toward a decrease in the levels of mRNA expression of the *PINK1* gene in HT. Herewith, the level of mRNA expression of the *Parkin* gene in HR did not change (Figure 3).

### 3.4. Immunoblotting Analysis of Heart Proteins of the Control and Hyperthyroid Rats

**Figure 4:**
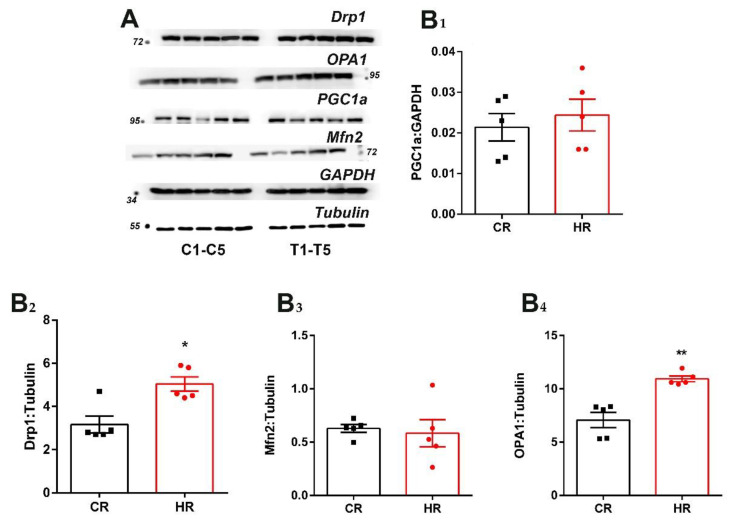
Immunoblotting analysis of the mitochondrial dynamics and biogenesis proteins in heart tissue of control and hyperthyroid rats: (**A**) representative Western blot of Drp1, OPA1, Mfn2, PGC1α, C_1_-C_5_-CR and T_1_-T_5_-HR; (**B**) relative levels of appropriate proteins with respect to the loading control (GAPDH or tubulin). CR, control; HR, hyperthyroidism. * *p* < 0.05 and ** *p* < 0.02, as compared with the control data (n = 5).

We also analyzed the level of the proteins responsible for mitochondrial biogenesis, dynamics and mitophagy in the investigated rats. In the hyperthyroid animals, the level of PGC1a and Mfn2 proteins did not change as compared with the control rats (Figure 4). At the same time, the level of OPA1 and DRP1 proteins increased by 1.6 times (Figure 4).

**Figure 5:**
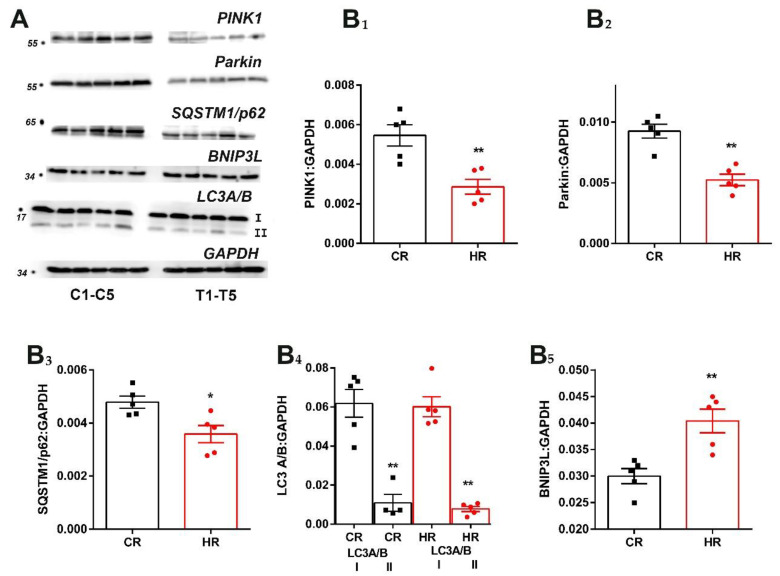
Immunoblotting analysis of mitophagy proteins in heart tissue of the control and hyperthyroid rats: (**A**) representative Western blot of Parkin, PINK1, SQSTM1/p62, BNIP3L, LC3A/B-I-II, C_1_-C_5_-CR and T_1_-T_5_-HR; (**B**) relative levels of the appropriate proteins with respect to the loading control (GAPDH). CR, control; HR, hyperthyroidism. * *p* < 0.05 and ** *p* < 0.02, as compared with the control data (n = 5).

Mitochondrial dynamics and mitophagy are the most important processes underlying mitochondrial homeostasis. One of the most well-investigated mitophagic mechanisms in mammalian cells is the PINK1–Parkin-mediated system [11]. In rats with hyperthyroidism, the PINK1 and Parkin levels decreased 1.9-fold (Figure 5). We also determined the levels of SQSTM1/p62 and LC3A/B, the proteins considered to be autophagy markers (Figure 5) [18]. The level of SQSTM1/p62 in HR heart proved to decrease by 1.3 times compared to this parameter in CR. Herewith, neither the total level of LC3A/B nor the level of its form LC3A/B-II, which binds to the autophagosome, changed in HR (Figure 5). In addition, we measured the levels of the BNIP3L protein, a receptor involved in a Parkin-independent form of autophagy. It turned out that the level of BNIP3L increased in HR by 1.4 times (Figure 5).

## 4. Discussion

Thyroid hormones are some of the most important regulators of physiological processes in higher organisms. Given their significant impact on energy metabolism and the fact that mitochondria are the main site of many metabolic transformations, these organelles have been and are the subject of key and extensive studies of various pathologies associated with excess or lack of TH. In this work, the state of hyperthyroidism was modeled by intraperitoneal administration of thyroxine (200 µg T_4_ per 100 g of animal weight for 7 days). The development of hyperthyroidism was indicated by severalfold increased concentrations of free T_3_ and T_4_ in the blood plasma of animals. As it was already said, there is no universal molecular model to explain the various actions of TH. Substances injected intraperitoneally enter the liver through the portal vein and only then reach systemic circulation. The liver, being a protective barrier, carries out the biochemical transformation of foreign and toxic compounds. It is the liver, unlike other organs, that is exposed to the strongest effects of the investigated substances during intraperitoneal administration. Thus, the results after the introduction of TH are likely to differ in the investigated animal organs, depending on the individual characteristics of the animal [12]. 

It is known that THs have the ability to enhance cellular respiration, in some cases with a simultaneous decrease in metabolic efficiency [1]. There are several mechanisms that explain the so-called calorigenic effect of TH:

1. The effect of TH on the uncoupling of mitochondrial respiration.
2. The effect of TH on the activity of mitochondrial respiratory chain complexes.

Earlier, we showed an increase in the level and activity of individual electron transport chain complexes (ETCs) in HR heart mitochondria; herewith, the activity of CII and CII+III decreased. Though the activity of most ETCs increased, the activation of respiration in HR heart mitochondria—usually observed in hyperthyroidism—has not been shown [19,20]. Under these conditions, an increase in the rate of respiration was observed only in state_4_ during the work of CI and CII; when using L-palmitoylcarnitine/malate as a substrate, the oxygen uptake by HR mitochondria decrease. Herewith, the respiratory control ratio decreased by 15–20% in HR in all cases [16]. Thus, it can be noted that both the uncoupling of oxidative phosphorylation and the disturbance of individual ETCs occurred in rat heart mitochondria in HT.

Mitochondria are capable of responding to changing energy requirements by remodeling their structure and shape, in particular the number and shape of cristae. Ultrastructurally, cristae are highly specialized areas where oxidative phosphorylation system complexes form and respiratory supercomplexes assemble [21]. The formation of such supercomplexes stabilizes individual complexes, enhances the flow of electrons between ETC enzymes and restricts the generation of reactive oxygen species, which is important for preserving the composition and content of lipids that support the structure of supercomplexes themselves [22,23,24,25,26]. All of the above advantages may not work or have a limited effect in various HT models, which are accompanied by the development of oxidative stress and an increase in membrane lipid peroxidation [16,27,28,29].

The analysis of heart tissue electron micrographs showed that cardiomyocyte mitochondria after HT induction featured various ultrastructural changes. More than half of the population of mitochondria were swollen, closely adjacent to one another and featured a partial disorientation of cristae (Figure 1D–F and Table 2). This fraction was dominated by organelles of increased size. In 13% of mitochondria, we observed a change in shape, emergence of vacuoles and local destruction of cristae (Figure 1G–I and Table 2). A displacement of myofibrillar mitochondria into the sarcolemmal zone of cardiomyocytes was noted. The swelling of mitochondria and the formation of large vacuoles in their matrix are the most common changes in the ultrastructural organization of mitochondria [30,31,32]. As for the MLBs occurring in HR heart mitochondria, there are studies demonstrating that the mitochondrial membrane material can be used to form a developing autophagosome both in the norm and in pathology [33]. Another interesting feature was the presence of a cluster of mitochondria located inside the HR nucleus. Earlier in the literature, it had been customary to consider mitochondria inside the nucleus as a consequence of diseases or pathological processes, or even as an artefact that emerges during the preparation and fixation of tissue. The current hypothesis is that the presence of functional mitochondria in the nucleus is not only a consequence of certain pathologies but rather represents a normal biological phenomenon involved in mitochondrial nuclear interactions (exchange of ATP/ADP, regulatory proteins and genetic material between organelles) [34,35].

The maintenance of the cell’s energy requirements can also be compensated for by enhancing mitochondrial biogenesis, which is activated by TH [36,37]. In this work, administration of T_4_ did not induce an increase in the biogenesis in heart tissue neither at the level of *Ppargc1a* gene expression nor PGC1a protein synthesis (Figure 3 and Figure 4).

The changes in mitochondrial metabolism under normal and pathological conditions attracted attention to two processes, mitochondrial dynamics and mitophagy, which were identified as key factors in mitochondrial quality control. It turned out that the expression of the *OPA1* and *Drp1* genes, as well as the level of these proteins, increased in HR (Figure 3 and Figure 4). OPA1, responsible for mitochondrial fusion, plays an important role in maintaining the structural organization and integrity of the inner mitochondrial membrane. As the number of mitochondria tended to decrease but the biogenesis process did not change, it is quite possible that fusion is required to optimize the mitochondrial function to enable the cell to cope with an increased energy demand in this HT model [38]. Interestingly, elongated mitochondria are spared from the engulfment by the phagophore and subsequent degradation. It is quite possible that this mechanism allows mitochondria to maximize the production of cellular energy and maintain cell viability during a lack of nutrients [39,40]. 

Thyroxine administration led to a decrease in the number of cardiac mitochondria in an area of up to 0.55 µm^2^ (Figure 2B). It is possible that the increased level of Drp1 is associated with the formation of mitochondrial vesicles, which occurs in animal cardiac tissue both under stress and under normal conditions, and is an important maintenance mechanism and the first line of defense against mitochondrial stress [41,42].

The study of mitophagy on this HT model showed unchanged expression of the *PINK1* and *Parkin* genes, but the level of these proteins decreased in HR heart (Figure 3 and Figure 5). The level of one of the autophagy markers, p62, decreased, and the other, LC3A/B-II, did not change in HR (Figure 3 and Figure 5). The canonical form of mitophagy was not activated in the HR heart; moreover, excess TH caused a decrease in the level of PINK1 and Parkin proteins probably at the stage of synthesis/posttranslational modification of the polypeptide chain. As for the decrease in the level of p62 in HT, it is not completely clear whether p62 is excreted exclusively by autophagy or whether the ubiquitin–proteasome pathway is also involved [43]. The determination of the level of BNIP3L, a noncanonical mitophagy receptor, showed that its amount increased in HR heart compared with CR.

Thus, in the HT state we observed the inhibition of PINK1/Parkin-dependent mitophagy in the heart of the investigated animals. On the other hand, the formation of MVs and an increase in the level of the noncanonical mitophagy receptor p62 were noted, which are, possibly, compensatory processes in maintaining intact homeostasis of HR heart mitochondria.

## 5. Conclusions

This paper showed that the mitochondrial quality control system reacted to changes under the influence of TH. The analysis of heart tissue electron micrographs showed abnormalities in the ultrastructure of HR mitochondria. The activation of the biogenesis process was not found using this HT model, but proteins responsible for changes in cardiac mitochondrial dynamics increased. The PINK1/Parkin-dependent mitophagy process was disrupted; at the same time, we noted the formation of mitochondrial vesicles and an increase in the level of the noncanonical mitophagy receptor p62, which can be the first line of defense in supporting the homeostasis of HR heart mitochondria.

## Figures and Tables

**Table 1:** List of gene-specific primers for the real-time PCR analysis.

Gene	Forward (5′→3′)	Reverse (5′→3′)
*Drp1*	GATCCAGATGGGCGCAGAAC	ATGTCCAGTTGGCTCCTGTT
*Mfn2*	AGCGTCCTCTCCCTCTGACA	TTCCACACCACTCCTCCGAC
*OPA1*	GCAGAAGACAGCTTGAGGGT	TGCGTCCCACTGTTGCTTAT
*PINK1*	GATGTGGAATATCTCGGCAGGA	TGTTTGCTGAACCCAAGGCT
*Parkin*	GGCCAGAGGAAAGTCACCTG	CACCCGGTATGCCTGAGAAG
*Ppargc1α*	TGACATAGAGTGTGCTGCCC	GCTGTCTGTGTCCAGGTCAT
*Actb*	GACCCAGATCATGTTTGAGACCT	CCAGAGGCATACAGGGACAAC

**Table 2:** T_3_ (free) and T_4_ (free) concentrations and body and heart weights in the control and hyperthyroid rats.

	CR	HR
T_3 free_, pmol/L	5.2 ± 0.1	9.3 ± 1.2 ***
T_4 free_, pmol/L	19.2 ± 1.0	66.2 ± 4.4 ***
Body weight, g	259 ± 2.3	235 ± 3.1 ***
Body weight gain, g	37 ± 2.0	15 ± 2 ***
Heart weight, g	0.9 ± 0.02	1.25 ± 0.03 ***
Heart/body weight (×10^3^)	3.5 ± 0.06	5 ± 0.13 ***

CR, control; HR, hyperthyroidism. *** *p* ˂ 0.01, as compared with the control data (n = 15 to determine T_3_/T_4_; n ≥ 40 to calculate body weight, heart weight, body weight gain, and heart/bodyweight).

**Table 3:** Morphometric parameters of heart mitochondria in the control and hyperthyroid rats.

	CR	HR
Number of mitochondria per image (48 µm^2^)	38.3 ± 3.5	30.3 ± 2.2
Number of swollen mitochondria relative to the total number of mitochondria, %	8	55
Number of swollen mitochondria with MLBs relative to the total number of mitochondria, %	-	3
Number of damaged mitochondria relative to the total number of mitochondria, %	2.5	13

The number of examined images in each group was approximately 50. Mitochondrial subpopulations occurred in the groups. The number of mitochondria analyzed in each group varied from 600 to 700. CR, control; HR, hyperthyroidism (n = 4 in each group).

## Data Availability

The data presented in this study are available upon request from the corresponding author.
